# From a Reflective Inquiry to a Proposed Revised Moral Formation of Current and Future Health Professionals

**DOI:** 10.5041/RMMJ.10586

**Published:** 2026-10-26

**Authors:** Shmuel P. Reis

**Affiliations:** 1 Digital Medical Technologies, Holon Institute of Technology, Holon, Israel; 2 Center for Medical Education, School of Medicine, Hebrew University, Jerusalem, Israel; 3 Clalit Health Services, Haifa and Western Galilee District, Gilon and Misgav Village Clinics, Israel

**Keywords:** Antisemitism in healthcare, history-informed professional identity formation, medical education, medicine, Nazism and the Holocaust, moral distress, moral education of the health professional

## Abstract

The *Lancet* Commission on medicine, Nazism, and the Holocaust (LCmNH) presented an innovation for the moral formation of health professionals. Proposing history-informed professional identity formation (HIPIF), it envisioned a morally resilient clinician who exercises moral agency, recognizes the fragility of medicine’s core values, pursues lifelong learning in medical ethics beyond principlism, and firmly addresses antisemitism, racism, and hate. Tragically, just before its publication (November 2023), the report gained greater relevance in the wake of the October 7, 2023, Hamas massacre, which led to a surge in antisemitism, including within healthcare and medical education. Unexpected barriers, such as fear of controversy, hampered dissemination of the LCmNH recommendations. Concurrently, the author, an Israeli Jew, physician, and Co-Chair of the LCmNH, grappled with moral distress in the context of the war following October 7 and antisemitism in healthcare. These challenges triggered a reappraisal of the LCmNH moral formation proposal. This reappraisal identified moral distress, antisemitism, and inadequate institutional accountability as central issues requiring attention. The author proposes a revised approach for moral formation, integrating mechanisms to prevent and identify moral distress and antisemitism, provide care for those affected, and pair systemic accountability with individual integrity. Steps for stakeholders to promote the updated approach, along with its expected benefits, are described. A diagram and comparative table illustrate and indicate areas of focus for the proposed enhanced approach. The author also shares how his reflective inquiry, scholarly pursuit, and reappraisal of the LCmNH report worked together to foster his own resilience and generate insights that informed the proposed updated approach to moral formation.

## INTRODUCTION: THE CURRENT MORAL LANDSCAPE OF CLINICAL WORK AND THE AUTHOR’S MORAL EXPERIENCE

In general, health professionals today practice in an environment marked by extraordinary progress, expanding expectations, and increasingly complex systems. Concurrently, disturbing phenomena exist that shape the moral landscape of clinical work, such as moral distress^1^–^6^ and the substantial rise in anti-semitism in healthcare and medical education.^7^–^13^

This morally turbulent landscape is also a source of my personal moral distress as an Israeli Jewish physician in the aftermath of the October 7, 2023, Hamas massacre.^14^ Coping with these challenging realities—including October 7 and its aftermath, rising antisemitism, and moral distress—has become a core preoccupation for me, now in my fiftieth year in practice, both as a person and as a professional, and triggered a reflective inquiry.

Since October 7, 2023, I have joined like-minded colleagues in addressing the growing international criticism of Israel and how, in some cases, it has morphed into the rise in antisemitism.^14^,^15^ Furthermore, in a previous paper, a colleague and I addressed moral issues stemming from Hamas atrocities and medical crimes, such as the killing of Israeli health professionals and holding, abusing, and killing hostages on hospital premises.^15^

Over this period, I have become increasingly aware of my own moral distress. While experiencing the Israeli healthcare system as upholding medical ethics under extreme circumstances,^16^ I also experience uncertainty about Israeli leadership’s motives and actions, profound sorrow over the suffering of non-combatants in Gaza, and apprehension that their enormous suffering is being inflicted “in my name.” This tension was recently aptly expressed by an Israeli ethicist and translated by the author:

“We, the citizens, have not decided to allow … to incessantly harass … Palestinians [in the West Bank] … we have not decided … to destroy innumerable buildings [in Gaza] … These have not been inflicted on my behalf or for my benefit. The opposite is true: they are against my conviction and my benefit.”^17^

In addition, a host of ethical dilemmas faced by Israeli health professionals, in and out of uniform, have arisen and persist, challenging those directly implicated, as well as others such as myself who teach, mentor, and counsel them.^18^

The confluence of these issues intertwines with my role as Co-Chair of the *Lancet* Commission on medicine, Nazism and the Holocaust (LCmNH).^19^,^20^ This Commission was charged with curating the historical evidence of Medicine’s involvement in Nazism and the Holocaust, examining its current implications for the profession, and developing relevant, history-informed moral formation curricula. The LCmNH published and launched its report in November 2023, coincidentally a month after the October 7 attacks,^14^,^15^,^19^,^20^ thrusting reality, personal struggle, and scholarly work into a complex and profound conflict. In addition, these circumstances quickly called for a reassessment of the LCmNH report.

The result of this professional work, alongside the personal reflective inquiry triggered by my own moral distress, is this essay. It explores moral distress^1^–^6^ and antisemitism in health settings,^7^–^13^ and their impact on clinician moral and professional identity formation. Further, the essay discusses ways in which structures, policies, training, and education must adapt, beyond existing paradigms and approaches, to prepare and support clinicians for ethical practice in a morally turbulent age. It does so through the lens of the 2023 LCmNH report,^19^,^20^ previous work on the role of medicine, Nazism and the Holocaust (mNH) in medical education,^21^,^22^ the impact and aftermath of the October 7 Hamas massacre,^14^,^15^ the global marked rise in antisemitism in healthcare,^7^–^15^ extensive scholarly consultation, and personal reflection on my own personal distress.

Integrating constructs and paradigms with practical issues—spanning personal and professional, local and global—I propose a revised approach that supports clinicians and guides their moral formation.

## PROFESSIONAL IDENTITY FORMATION AMID MORAL DISTRESS AND ANTISEMITISM

First described in nursing literature, *moral distress* was defined by Jameton in 1984 and cited in his 2017 publication as “knowing the right thing to do while being in a situation in which it is nearly impossible to do it.”^4^ This definition was further developed into the concept of the psychological suffering that arises when clinicians know the ethically appropriate action to take, but are constrained in acting by institutional policies, hierarchical structures, legal frameworks, resource limitations, lack of support, or increasing clinical complexity within challenging contexts.^5^,^23^ Today, this is referred to as constrained moral distress.^1^–^6^,^23^–^27^ Moral distress may also arise when clinicians are uncertain about the appropriate moral course of action, expanding its definition to include *moral uncertainty*.^5^,^23^–^27^ Unlike *moral dilemmas*— where competing values must be weighed and an ethically sound decision taken—moral distress occurs both when the ethically correct path is clear, but blocked, and when uncertainty prevails (such as during the pandemic).^1^–^3^,^5^,^23^ Uncertainty may result in a sense of powerlessness, guilt, and erosion of moral integrity.^24^,^26^,^28^ In addition, moral distress can result from witnessing an ethical transgression, without committing it.^6^ An additional relevant construct is *moral injury*:

Moral injury can arise where sustained moral distress leads to impaired function or longer-term psychological harm. Moral injury can produce profound guilt and shame, and in some cases also a sense of betrayal, anger and profound “moral disorientation”. It has also been linked to severe mental health issues.^6^^(p3)^

As healthcare systems grow more bureaucratic, technologically mediated, and resource-constrained, and as geopolitical forces such as war, racism, pandemics, and climate change intrude, moral distress has become a defining feature of clinicians’ professional lives.^2^–^6^,^23^ For example, technology has introduced additional ethical challenges such as data security,^29^ and physicians in uniforms with dual loyalties encounter situations that trigger moral distress. 30 A recent study examined the moral distress experienced by third-year medical students at a US institution during their transition to clinical training.^31^ Of the 407 vignettes examined, students agreed (*n*=140; 34.4%) or strongly agreed (*n*=30; 7.4%) that they experienced moral distress in a total of 170 (41.8%) cases. Moral distress and injury significantly pervade the entire professional life cycle.^3^,^32^,^33^ However, this is not merely an individual psychological burden; it also constitutes a structural and systemic issue (see *Proposed Revised Approach for Moral Formation of Health Professionals*, below).^24^,^27^,^28^,^31^–^33^

Moral distress is compounded when healthcare professionals face discrimination against themselves, challenging wellbeing, ethical and existential resilience, and ability to provide equitable care.^12^,^13^ For Jewish clinicians, antisemitism has been a longstanding burden. It is defined by the International Holocaust Remembrance Alliance (IHRA) as “a certain perception of Jews, which may be expressed as hatred toward Jews. Rhetorical and physical manifestations of antisemitism are directed toward Jewish or non-Jewish individuals and/or their property, toward Jewish community institutions and religious facilities.”^34^ Antisemitism is extensively addressed in current literature,^7^–^13^ including in *Rambam Maimonides Medical Journal*.^10^ For example, Wald and Roth delineate education, engagement, empathy, and enforcement to help overcome a hostile learning environment in educational institutions.^7^ They propose countering antisemitism with intervention programs and point out that excellent resources are already available for countering antisemitism.^7^

Moral distress and discrimination in healthcare not only threaten clinicians’ wellbeing but also their professional identity. Professional identity formation (PIF)^35^–^38^ is “a representation of self, achieved in stages over time during which the characteristics, values, and norms of the medical profession are internalized, resulting in an individual thinking, acting, and feeling like a physician.”^35^^(p1447)^ When professionals repeatedly act against their moral convictions, they may experience a loss of integrity, purpose, and meaning.^1^–^6^,^23^–^27^ In the Jewish or Israeli context, when clinicians need to hide their Jewish or Israeli identity and feel unsafe or intimidated, both their wellbeing and identity are compromised.^13^,^31^,^33^

Moral distress, the rise in antisemitism, and their resulting consequences on professional identity and moral formation underscore the need for moral education that supports the development of a resilient and grounded professional identity. Operationalizing this need, the LCmNH developed a new construct: history-informed professional identity formation (HIPIF),^19^,^20^ defined as “the shaping of professional identity through learning about, and reflecting on, historical episodes in which medical values and priorities were challenged.”^19^^(p1868)^ The core of HIPIF is cultivating moral agency, challenging learners to question professional structures and culture (or proposed changes to them), to “critically scrutinize them, explore their origins, assess the alignment between one’s personal values and those of the profession, and examine both for areas of weakness.” 19^(p1913)^ Through its report and medical education appendix, the LCmNH proposes a detailed scholarly and practical roadmap for rendering mNH education a core component of the moral development of health professionals. It is further described in the upcoming section.

## THE *LANCET* COMMISSION ON MEDICINE, NAZISM, AND THE HOLOCAUST, AND MEDICAL ETHICS EDUCATION

Historically, moral education in the health professions has centered on bioethical principles (autonomy, beneficence, non-maleficence, distributive justice^39^); case-based reasoning;^40^ professional codes of conduct;^41^ legal and regulatory frameworks;^41^,^42^ and virtue ethics.^43^ These remain essential, providing a shared language for ethical deliberation. The following discussion focuses on selected limitations of conventional approaches rather than characterizing medical ethics education as a whole. However, the traditional bioethics approach often assumes that clinicians have the freedom to act on their moral judgments. In the age of rising antisemitism and moral distress, this assumption may no longer be valid.^1^–^13^ Traditional bioethics education often emphasizes idealized scenarios, while clinicians face messy, ambiguous, and constrained realities.^18^,^44^ It teaches what ought to be done, but not how to cope when what ought to be done cannot be done or involves significant uncertainty.^1^,^2^,^5^,^18^,^44^

The 2023 LCmNH report highlights that while foundational ethics instruction remains necessary, extending and integrating existing approaches would enhance support for moral development and navigating ethical challenges.^19^ Such challenges emerged significantly during the pandemic, where crucial moral dilemmas prevailed, while practitioners experienced a lack of timely, relevant ethical guidance.^2^,^5^,^44^,^45^ The report contends that ethics education may benefit from being supplemented with newer forms of moral education that address the emotional, relational, and structural dimensions of ethical practice, including the often ignored gap between the moral ideal and the moral reality.^5^,^7^,^18^,^27^–^30^,^45^,^46^ This gap, described by Kleinman, is between what clinicians believe in as their moral ideal and the daily reality they face and experience, which is invariably and significantly different from the ideal.^46^ Moral distress often stems from this gap, and learning to contain, acknowledge, and continue to morally evolve while facing this duality is an essential developmental challenge for clinicians.^46^

In order to address the full scope of professional bioethics, including moral distress and burnout, relevant educational interventions abound,^45^,^47^–^49^ such as the proliferation of diversity, equity, and inclusion curricula in mostly North American health professions education institutions.^50^ Contributing to this effort, the LCmNH report introduced an innovation to the moral education of health professionals, calling for medicine, Nazism and the Holocaust (mNH) education to be required in health professions education, throughout the entire professional life cycle.^19^,^20^ It proposes that “learning about, and reflecting upon, this history supports the formation of morally courageous and resilient professionals … and of a professional community with greater capacity to shape and support moral courage and resilience.” 19^(pp1911–12)^

Drawing on the 2010 *Lancet* Commission on Education of Health Professionals for the 21st Century^51^ framework for health professions education, the 2023 LCmNH report states that “beyond an informational level of learning, education centered on this history can also result in learning on the formational and transformational levels.”^19^^(p1867)^ This reflects an earlier Commission’s view that transformative learning^51^–^53^ aims to develop leadership attributes and produce “enlightened change agents.”^51^^(p1924)^

The report’s launch coinciding with the October 7, 2023, Hamas massacre and its aftermath led to a published statement addressing these events, which highlighted the report’s relevance to fighting antisemitism and hate of all kinds.^15^

Evidence of the positive impact of mNH learning on personal and professional identity formation, including moral formation, has been published. Benefits demonstrated include greater moral courage and increased awareness of biases and inequities in healthcare, among others, with the historical context amplifying impact.^20^,^54^–^58^

Despite the demonstrated contribution of mNH, the increasingly turbulent moral landscape of clinical practice, rising antisemitism and moral distress, and the unexpected barriers to disseminating and implementing the LCmNH recommendations prompted a reappraisal of its proposed approach to moral education. How could this approach be adapted to these challenges, particularly when fear of controversy has made educators reluctant to offer mNH education, let alone make it mandatory and allocate more curricular time to it?^14^,^59^,^60^

## PROPOSED REVISED APPROACH FOR MORAL FORMATION OF HEALTH PROFESSIONALS

The recent rise in antisemitism and its omission from diversity, equity, and inclusion discourse have engendered criticism and hampered the progress of moral education innovations, including the LCmNH recommendations.^7^,^14^,^50^,^61^

A second trigger for the reappraisal was the emergent realization that moral education should not be the responsibility of individual educators alone. Indeed, individual moral education remains needed: medicine has been described as a “moral enterprise” 62 with profound effects on all aspects of being. Yet, a waning of moral reasoning,^63^–^66^ and moral segmentation—described as “a fracturing of moral competence in a new and bewildering context,”^65^^(p5)^ an early moral development stage typical for medical students—are reported during and after medical school. In this context, the LCmNH report proposed that learning about the history of mNH may initiate or support “an educational focus on empathy, compassion, moral reasoning, and justice in caregiving.” 19^(p1912)^ However, what was not emphasized enough in the LCmNH report was institutional responsibility.

In reassessing the LCmNH report, I found a striking lack of institutions demonstrating ethical structural competence, creating environments that support ethical practice, reducing unnecessary moral distress, and fighting antisemitism.^5^,^27^–^28^,^67^–^71^ This led to an understanding that when institutions fail to take responsibility for and address systemic problems—such as moral distress, antisemitism, poverty, or healthcare inequity—moral education becomes a form of moral burden-shifting. Instead of authorities providing systemic solutions, it places blame on individuals for problems they did not create and assigns them responsibility to cope with those same sinister issues through personal lifestyle, mindset, or behavioral changes.^72^–^74^

As Molendijk warns, “… paradoxically, these ethics measures may increase the risk of moral injury by shifting the moral burden entirely onto frontline workers.”^73^^(p4)^ Clinicians who understand the system are better equipped to challenge unethical constraints, propose reforms, and participate in institutional decision-making.^67^,^68^,^75^–^77^ This knowledge empowers them to transform moral distress and discrimination into constructive action.^7^,^68^,^77^ They also acquire moral resilience, the capacity to preserve or restore integrity in response to moral adversity. Resilience is not about suppressing distress or accepting unethical conditions; rather, it involves cultivating the skills and dispositions needed to navigate moral complexity without losing one’s sense of purpose, agency, and meaning.^7^,^77^

The proposed revised approach places mNH learning at its core and incorporates training in mindfulness, communication, and conflict resolution—skills that help clinicians remain grounded and effective when facing moral adversity.^78^,^79^ It also addresses the systemic forces that shape moral agency.^5^,^27^,^28^,^67^

A noteworthy dimension in addressing moral distress and antisemitism is that these challenges are often intensified by isolation. Clinicians may feel alone in their concerns, unsure whether others share their existential and ethical discomfort. By creating developmental spaces for open and interprofessional dialogue, institutions can transform moral distress and exposure to antisemitism from a private burden into a shared opportunity for learning and advocacy.^32^,^33^ Ethical spaces and communities help clinicians recognize that experiencing moral distress or racial discrimination does not represent a personal failure but a systemic signal that something needs attention.^1^–^3^,^19^,^23^,^27^–^28^,^32^,^33^,^76^,^79^

In reappraising the LCmNH report, I connected present-day antisemitism in healthcare and medical education, the health professionals’ moral distress crisis, and the history-informed lessons of mNH. With scholarly consultation, I developed a comparative table (Table 1) and conceptual diagram (Figure 1) to illustrate the proposed approach at the individual, professional, and institutional levels. Table 1 contrasts selected limitations of conventional approaches with the proposed enhancements, while Figure 1 traces the progression from current challenges to intended outcomes. Both constitute a representation of the approach of change proposed in this essay. Practical steps for implementing this enhanced approach are proposed in the next section.

**Table 1:** Comparison of Conventional and Proposed Enhanced Approaches to Moral Formation in Health Professions Education.

Dimension		Selected Limitations of Conventional Approaches	Proposed Enhanced Approach
**1**.	**Focus**	*Adherence and Liability Avoidance* Medical ethics is generally framed as adherence to the four principles (autonomy, beneficence, non-maleficence, justice) and to institutional rules, with an emphasis on liability avoidance.	*Moral Agency and Resilience* Lifelong cultivation of moral agency and resilience, acknowledging moral emotions and care, upholding human rights, and resisting ethical erosion in ambiguous, high-pressure environments.
**2**.	**Core theoretical framing**	*Cognitive Dilemma Analysis* Analyzing idealized case studies using normative bioethical theories; viewing ethical choices as separate from personal identity, emotions, relations, narrative, or historical context.	*Context-Informed Deliberation* Integrating personhood and context, moral emotions, relations, and structural issues. Leveraging historical lessons (e.g. medicine under Nazism), mindfulness of the fragility of professional ethics, and potential corruption of moral agency by coercion or temptation.
**3**.	**Pedagogical approach**	*Didactic and Idealized* Lectures, guidelines, and standard policy awareness-training focusing on scenarios addressing “what ought to be done” while often ignoring real-world constraints of “what can be done.”	*Experiential, Reflexive, and Transformative* Active, reflective dialogue (ethics debriefings, narrative medicine, reflective journaling), integrating the *moral ideal*–*moral reality* gap and building moral resilience/courage. This approach aims to transform personal moral distress into collective advocacy and systemic reform.
**4**.	**Scope of discrimination and anti-bias training**	*Selective Diversity Curricula* Diversity, equity, and inclusion and antibias efforts that remain relatively abstract, non-universal, or overlook specific forms of hate, such as rising antisemitism in health settings.	*Comprehensive Structural Responsibility* Mandatory, historically grounded training that explicitly equips clinicians to confront antisemitism, racism, and all forms of discrimination as core professional responsibilities.
**5**.	**Understanding of moral distress**	*Individualized Burden and “Bad Actors”* Moral failure attributed to personal wrongdoing or ignorance; moral distress treated as a private psychological burden or individual coping defect (moral burden-shifting).	*Systemic and Structural Dysfunction* Institutions recognize moral distress and discrimination as systemic signals, addressing structural drivers, bureaucratic constraints, and power dynamics. Individual moral development is paired with institutional accountability.

Note: The conventional approach represents selected characteristics and limitations of existing medical ethics education rather than a comprehensive description of all current educational practices. The proposed enhanced approach reflects the conceptual model advanced in this article.

**Figure 1:**
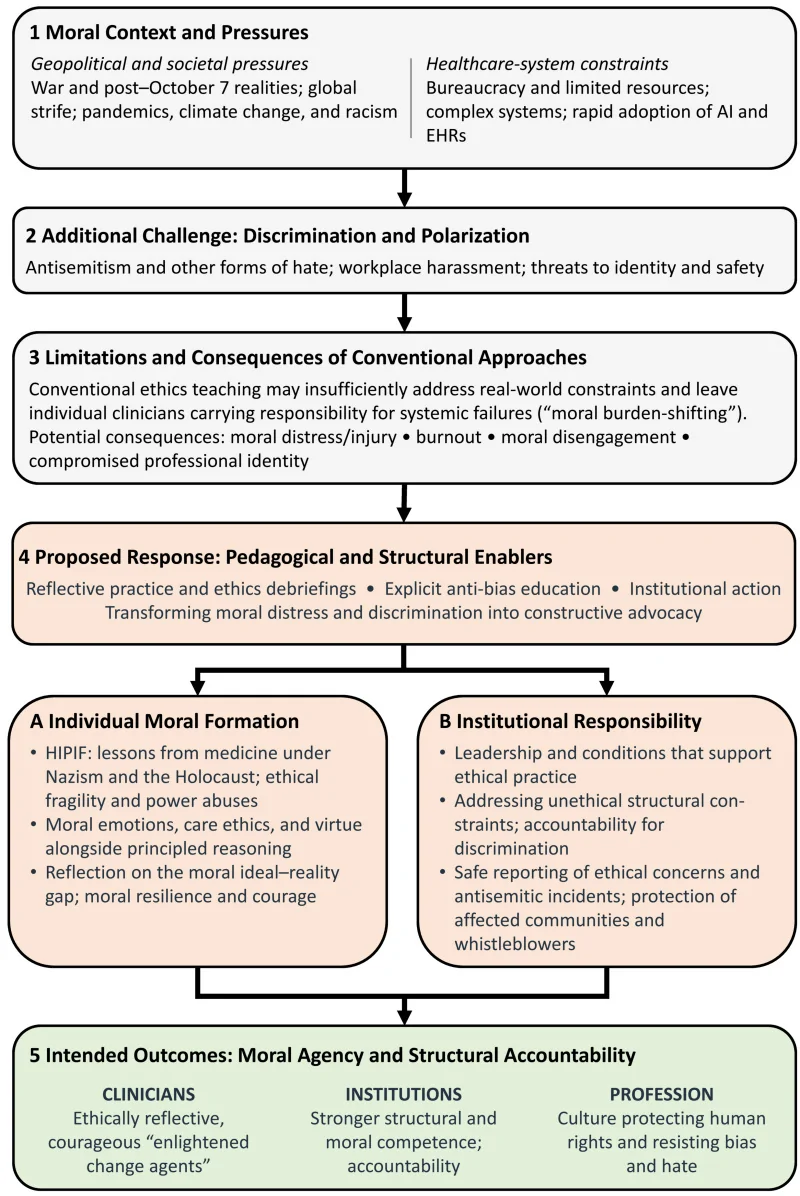
Proposed Model Approach for Moral Formation in Healthcare Integrating Individual Development with Institutional Accountability. Note: Grey boxes represent the current context, challenges, and limitations of existing approaches; orange boxes represent the proposed educational and institutional changes; and the green box represents the intended outcomes. AI, artificial intelligence; EHRs, electronic health records; HIPIF, history-informed professional identity formation.

## ROADMAP FOR IMPLEMENTATION AND DISSEMINATION

During a webinar by the Association of American Medical Colleges on the LCmNH report (January 25, 2024), the *Lancet* editor-in-chief aptly called the report “a manifesto for action.”^58^ The actions called for include addressing antisemitism, rethinking the moral education of health professionals, and transforming the structural and systemic components of healthcare and medical education.^19^ However, efforts to disseminate the report and promote wider adoption of mNH education in health professions programs appear to have made less progress than hoped for, but still significant.^80^ Although several examples of implementation are encouraging,^81^–^84^ other efforts have met with growing reluctance among leaders and learners to engage in mNH education because of fears of controversy amid heightened antisemitism following October 7, 2023.^59^,^60^ The report could not be launched in the United States, Canada, and the United Kingdom, as local champions felt it would be too disruptive. Successful launches were nevertheless held after October 7 in Vienna, Austria;^80^ Berlin, Germany; and Akko, Israel.

The conceptual revision proposed in the comparative table is further illustrated in Figure 1. Boxes 1–3 focus on the root causes and context of current moral challenges in healthcare and on existing approaches to clinician moral formation—as discussed earlier. To recap, current approaches to the moral formation of health professionals are based on principled ethics and focus on the individual. They emphasize moral judgment and justice (mastery of rules and principles); may benefit from real-time, actionable moral guidance in time-sensitive, high-pressure crises; and engage in moral burden-shifting^72^–^74^—in essence implying that systemic failure is a personal coping deficit. The result is a crisis with ubiquitous moral distress and moral injury; dehumanization of patients and peers; moral segmentation (splintered integrity);^65^ vulnerability to unchecked bias and hate; and widespread professional burnout and moral disengagement.

The pedagogical and structural changes^67^,^74^,^79^,^85^–^91^ that enable the implementation of this moral formation approach include reflective practice^84^ and debriefings (providing opportunities to share individual distress collectively); institutional accountability^67^,^86^–^88^ and safe spaces that challenge moral burden-shifting;^72^–^74^ and explicit integration of antibias education to confront antisemitism and hate.

Another important dimension for implementation of this approach is helping clinicians acknowledge, contain, and live within the gap between moral ideals and clinical reality,^46^,^47^ while cultivating moral resilience and active agency without cynicism.^79^,^89^

Most importantly, the revised LCmNH approach proposed here insists on integrating individual formation with systemic and structural changes.^67^ This also requires challenging unethical constraints and embracing controversy.

Practical steps and priorities for implementing this proposal include:

1. Structural support for ethical practice, such as adequate and equitable staffing and resources;^62^,^87^ transparent decision-making processes;^88^ supportive leadership that values ethical concerns and is committed to rooting out all expressions of hate;^86^,^89^ mechanisms for reporting and addressing both ethical issues and antisemitic incidents;^90^,^91^ and policies that protect affected communities and whistleblowers and encourage moral courage.^90^,^91^
2. The key components of moral resilience identified in the scholarly literature may serve as learning outcomes for educational interventions. These include self-awareness (recognizing one’s moral values, emotional responses, and limits);^79^,^89^,^92^ moral courage (the willingness to speak up or challenge unethical practices);^19^,^89^,^93^ adaptive coping strategies (managing distress without dis-engagement or cynicism);^8^ reflective practice (examining experiences to learn and grow ethically); 85 and boundary-setting (understanding when to advocate, when to compromise, and when to withdraw).^78^
3. Educational strategies that may help address moral distress include ethics rounds where teams discuss challenging cases;^92^,^94^ moral distress debriefings after ethically troubling events;^94^ interprofessional ethics training to foster mutual understanding;^76^ peer support groups that normalize moral struggle;^62^,^93^ and narrative medicine practices that encourage storytelling and meaning-making.^85^ These strategies are becoming increasingly available but are still far from being universal.^61^,^92^–^94^
4. The reappraisal also reinforces and updates key aspects of PIF emphasized in the LCmNH report, including articulating core values; integrating personal morality with professional norms; navigating value conflicts without losing authenticity; developing a sense of moral agency and pride in one’s religious and ethnic identity; and reinforcing the idea that moral struggle and fighting against discrimination are a normal part of professional growth.^19^

The proposed approach is broadly applicable to medical ethics education and guidance, and the fight against antisemitism. It may also assist in addressing racism, discrimination, and other forms of hate. At the core of this approach is learning from mNH and the history of antisemitism, which may be the implementation strategy of choice for the present approach.

While informed by the work of the LCmNH and additional scholarly consultation, the approach presented in this essay is the author’s own proposal. Unlike the existing recommendations for implementing mNH education, for which empirical evidence is available, the proposed approach has not yet been meaningfully applied, thus its validity and utility remain to be empirically evaluated.

## PERSONAL MORAL DISTRESS: TAKING STOCK

Once I recognized my own moral distress, I began coping through reflective inquiry, scholarly pursuit, and proactive efforts to address the challenges discussed in this essay. This process led to the conceptual insights described here and gave me a sense that these efforts have helped prevent my moral distress from escalating to moral injury.^6^ After over a year of experiencing moral disorientation, coupled with anger and a sense of betrayal, I still experience distress but am fully functional. The causes of my moral distress (and those of my colleagues) are still present, as no overall resolution of the geo-political situation and the suffering it engenders is in sight. For now, continuing to practice and reflect, engaging in learning, teaching, and advocacy, and acknowledging nuance and complexity remain essential to me, as do deep conversations with colleagues— including the Israel Defense Forces medical corps cadets and serving physicians—and experts.

My response has shifted from a justified reactive mode to what may be a more effective, proactive one, including in mitigating moral injury. This shift may represent another important aspect of coping with my ongoing existential moral distress.

I offer the proposed approach as reasoned invitation to change the moral formation of health professionals.

## CONCLUSIONS AND THOUGHTS FOR THE FUTURE

This fresh perspective on the moral development of health professionals provides a framework for grappling with both my professional and personal quest. Achieving fuller resolution may take time as events unfold, hopefully bringing an end to the current strife and tragedies. In the interim, accountability, advocacy, and proactivity must be upheld, moral distress acknowledged and addressed, and a commitment maintained to the fight against antisemitism. Although this paper draws attention to antisemitism in particular, the mNH curricula are crucial for combating all kinds of hate and discrimination.

There is a consensus among relevant scholars and stakeholders on the importance of innovation, reflective practice, and attention to identity and moral development.^19^,^20^,^35^–^38^,^41^–^43^,^45^,^47^,^85^ Despite current events posing challenges, significant implementation of the LCmNH recommendations is underway. My proposed approach, which builds on the LCmNH’s vision of transformative medical education aimed at developing “enlightened change agents,”^19^^(p1913)^ may offer a way forward.

Present and future leaders and practitioners who act with moral courage may experience profound personal and professional transformation and contribute to systemic changes within healthcare and health professions education, thus becoming better equipped to confront inevitable future challenges. There is a need to face controversy, rather than avoid it,^14^,^18^,^59^,^60^ and to eliminate moral burden-shifting once it is recognized.^72^–^74^ Let us hope that clinicians and their institutions confronting moral distress and antisemitism will take stock, gain awareness of the sources of both, and seek ways to turn the crisis into constructive action.

Emphasizing the prevention, identification, and healing of moral distress is vital, particularly at a structural level, and must become central to health professions education, medical ethics, and PIF. Addressing the rise of antisemitism in healthcare and medical education is intertwined with this focus. The ethos and competence of health professionals, as recommended by the LCmNH, need to include the responsibility to combat antisemitism, racism, and other forms of discrimination. The synergy of these initiatives can potentially transform healthcare, enhance our global citizenship, and deepen our sense of shared humanity.

I invite readers to join like-minded scholars and professionals in making this transformation a reality and hope that the ensuing conversation will be fruitful as we collectively strive to find light at the end of the dark tunnel of our times.

## Abbreviations

HIPIF: history-informed professional identity formation
LCmNH: Lancet Commission on medicine, Nazism and the Holocaust
mNH: medicine, Nazism and the Holocaust
PIF: professional identity formation
